# The RNA Editing Pattern of *cox2* mRNA Is Affected by Point Mutations in Plant Mitochondria

**DOI:** 10.1371/journal.pone.0020867

**Published:** 2011-06-13

**Authors:** Benoît Castandet, Alejandro Araya

**Affiliations:** Laboratoire de Microbiologie Cellulaire et Moléculaire et Pathogénicité (M.C.M.P.- UMR5234). Centre National de la Recherche Scientifique and Université Bordeaux Segalen. Bordeaux, France; The Rockefeller University, United States of America

## Abstract

The mitochondrial transcriptome from land plants undergoes hundreds of specific C-to-U changes by RNA editing. These events are important since most of them occur in the coding region of mRNAs. One challenging question is to understand the mechanism of recognition of a selected C residue (editing sites) on the transcript. It has been reported that a short region surrounding the target C forms the *cis*-recognition elements, but individual residues on it do not play similar roles for the different editing sites. Here, we studied the role of the −1 and +1 nucleotide in wheat *cox2* editing site recognition using an *in organello* approach. We found that four different recognition patterns can be distinguished: (a) +1 dependency, (b) −1 dependency, (c) +1/−1 dependency, and (d) no dependency on nearest neighbor residues. A striking observation was that whereas a 23 nt *cis* region is necessary for editing, some mutants affect the editing efficiency of unmodified distant sites. As a rule, mutations or pre-edited variants of the transcript have an impact on the complete set of editing targets. When some Cs were changed into Us, the remaining editing sites presented a higher efficiency of C-to-U conversion than in wild type mRNA. Our data suggest that the complex response observed for *cox2* mRNA may be a consequence of the fate of the transcript during mitochondrial gene expression.

## Introduction

RNA editing challenges the central dogma of molecular biology as it changes the information encoded for by the gene at the RNA level. This process affects a wide variety of organisms through nucleotide insertion/deletion or nucleotide conversion [1]. In plant mitochondria, RNA editing occurs by site-specific deamination of cytosines [2]. While around 30 editing events occur in the chloroplast transcriptome [3], more than 400 cytosine residues are changed to uracil in flowering plant mitochondria [4], [5], [6], [7], [8]. In most cases, changes occur in the coding region of mitochondrial mRNAs, increasing the similarity of the encoded proteins with their counterparts from organisms that do not edit their transcripts. Therefore, RNA editing constitutes an essential step to ensure the production of functional proteins and the proper functioning of mitochondria [9], [10].

One major issue is to understand how the editing machinery can recognize specific C residues on the transcript, since there is no obvious consensus sequence when comparing the different *cis*-elements on the transcriptome. Based on *in vitro* and *in organello* experiments, it has been reported that the *cis*-acting elements necessary and sufficient for recognition reside in a short sequence of fewer than 30 nucleotides encompassing the C target [11], [12], [13], [14]. However, in some cases, distal regions can affect the editing efficiency [15], [16].

Little is known about the *trans*-elements constituting the RNA editing machinery in plants. Organellar RNA editing *trans* factors were first discovered for the chloroplast [17] and recently, several PPR proteins have been identified as *trans* acting factors involved in mitochondrial RNA editing [18], [19], [20], [21], [22], [23], [24], [25], [26], [27], [28]. Since no deaminase activity has been found associated with these PPR proteins, it is postulated that they are involved in editing site recognition. The specific binding to the *cis* elements is thought to recruit the catalytic element, the cytosine deaminase. However, to date, specific binding to the *cis* element has only been reported for two PPR *trans* factor, CRR4 in the chloroplast [29] and PpPPR_71 in the mitochondria from the moss *Physcomitrella patens_* [24]. It should be noted that some PPR proteins participate in editing several C residues [19], [20], [23], [25], [26], [27], [28]. Considering that the *cis*-acting elements of these sites present no similarity, the mechanism allowing the recognition of different sites by *trans*-acting factors remains an important question.

To gain insight into the RNA editing process, we studied the consequences of point mutants of *cis* recognition elements on the editing capacity of *cox2* editing sites. Extensive mutagenesis analyses of C77 and C259 editing sites from wheat *cox2* demonstrated that although the extent of *cis*-elements is similar, the role of each residue is different in the recognition of these targets [11], [12]. Notably, two different recognition patterns were observed based on the requirement for either the +1 or the −1 neighbor nucleotide to the target C residue [11]. These observations have been confirmed by *in vitro* and *in silico* analyses [30], [31], [32]. One question is whether the identity of the nearest neighbor nucleotide of the editing site reflects a general recognition mechanism for *trans*-acting factors. In this case, selected residues in the *cis*-element should make it possible to discriminate between different editing sites on the transcript. Bioinformatic analyses suggest that *trans* acting factors may be able to distinguish purines from pyrimidines and, at particular positions, one specific nucleotide [33].

We present here the editing status of mitochondrial mRNAs from single and multiple mutants of *cox2* introduced into purified mitochondria. This approach allows the coincidental analysis of all editing sites on the same transcript. Our study reveals at least four different recognition patterns for C-to-U editing based on the importance of the contiguous residue. Furthermore, our findings suggest that RNA editing in plant mitochondria is not isolated but is rather part of a more general maturation process.

## Materials and Methods

All plasmids used in this study are based on the pCOX2Ta vector previously described [12]. They contain the inverted repeat region from the wheat *cob* gene (*Ir-cob*) (accession no. AF337547). This sequence combined with the 23 bp upstream insert sequence served to specifically amplify transgenic products with nested PCR.

### Construction of mutants

The list of the oligonucleotides used is given in Table S1. The strategy is based on the QuikChange® Site-Directed Mutagenesis Kit (Stratagene). Complementary primers bearing the mutation were used for PCR reactions on 100 ng of pCOX2Ta plasmid or derivatives. The PCR mixture contained 1 µM of each primer, 200 µM of each dNTP and 2.5 units of *Pfu* DNA polymerase (Stratagene) in a final volume of 50 µl, according to the supplier's protocol. Parameters for amplification were 95°C for 2 min, 20 cycles at 95°C for 30 s, 48°C for 30 s and 68°C for 14 min, and finally 68°C for 10 min. After amplification, 10 units of *DpnI* endonuclease (Promega) were directly added to the PCR reaction for 3 hours at 37°C to eliminate the original DNA template. Ten microliters of the digestion reaction were used to transform competent *E. coli* DH5α. The sequence of selected mutants was verified before use.

### Mitochondrial purification and electroporation

Mitochondria were prepared from 20 grams of wheat embryos essentially as described [34]. Sucrose gradient purified mitochondria were used immediately in electroporation experiments after protein determination using the Bio-Rad Protein Assay (Bio-Rad).

Electroporation was carried out with 1 mg of mitochondrial proteins in 50 µl of 0.33 M sucrose and 2 µg of recombinant plasmid in the conditions previously described [34]. Electroporated mitochondria were incubated for 18 h at 25°C with shaking at 130 rpm in a reaction mixture containing 0.33 M mannitol, 90 mM KCl, 10 mM MgCl_2_, 12 mM Tricine (pH 7.2), 5 mM KH_2_PO_4_, 1.2 mM EGTA, 10 mM sodium succinate, 1 mM GTP, 2 mM ADP, 0.15 mM (each) CTP and UTP, 2 mM dithiothreitol. Mitochondria were recovered by centrifugation at 15000× g for 15 min at 4°C. RNA was purified with 800 µl Trizol® reagent (Invitrogen) according to the supplier's protocol.

### RT-PCR

One microgram of nucleic acids obtained after Trizol® treatment was digested with 2 U of DNase I Amplification grade (Invitrogen) for 15 min at 25°C. cDNA synthesis was performed with 200 units of Superscript II RT (Invitrogen) using 100 ng of random primers hexamers as primers (Promega). PCR amplifications were performed with primers 1a and 1b using the Advantage 2 polymerase mix (Clontech) as follows: 95°C for 2 min, 20 cycles at 95°C for 30 s, 64°C for 1 min and 68°C for 2 min, and finally 68°C for 10 min. Primers 2a and 2b were used for nested PCR on 2 µl of PCR1. Parameters were the same for PCR2, except for annealing temperature and cycling, which were 55°C and 20 cycles respectively.

### Primers used in RT-PCR analyses

1a: GCGGTGCAGTCATACAGATCTGC

1b: TCCCGCGGGAAGCGGAAAGC

2a: GAGCAGAGCTGAAAAAGATG

2b: TATCCAGATTTGGTACCAAA

### Quantization of RNA editing

To determine the profile and the rate of C-to-U conversion in RT-PCR products, PCR bands corresponding to the mature transcripts were excised from the agarose electrophoresis gel and purified with the GFX PCR DNA and Gel Band Purification Kit (GE Healthcare). The purified fragments were ligated into pGEM-T easy vector as recommended by the manufacturer (Promega). Cloned PCR products were sequenced with the BigDye® Terminator Cycle Sequencing Kit v 1.1 (Applied Biosystems). Sequences analyses were performed at the Genotyping and Sequencing Facility of Université Bordeaux Segalen. Editing efficiency was determined by sequencing at least 16 cDNA clones from each electroporation experiment as described [35]. Editing efficiency variations in mutants between independent experiments were lower than 10%. Moreover, no significant variations in the ratio precursor/mature mRNA were observed.

## Results

The wheat *cox2* construct and their mutant derivatives are formed by two exons of 388 and 392 bp, split by a 1223 bp intron. The *cox2* transcript possesses 17 editing sites, 6 in the first and 11 in the second exon respectively [36]. After introduction of the recombinant DNA into *T. aestivum* mitochondria, the mRNA was analyzed by RT-PCR and the identity of spliced molecules was verified by sequence analysis. The editing efficiency of wild-type and mutant *cox2* transcripts was determined by sequencing at least 16 individual RT-PCR clones from each electroporation experiment. The results presented correspond to the percentage of C-to-U conversion for every editing site in the population of cDNA analyzed. The editing efficiency of site C259, which has been extensively studied [11], [12], was used as an internal standard and was systematically monitored for each assay. In all the experiments presented in this study, site C259 was edited with an efficiency higher than 85%.

### Four different recognition patterns exist based on +1/−1 dependency

RNA editing of sites C77 and C259 on *cox2* transcripts is known to be affected by mutations at the −1 and +1 residues, respectively [11], [12]. We extended these analyses to the entire transcript by mutating the neighbor −1 or +1 residue from the different *cox2* editing sites. The A residues were replaced by T and vice versa; the C residues were replaced by G, and the G residues were replaced by T. Site C385 belonging to the IBS1 region of exon1 was not modified.

**Figure 1:**
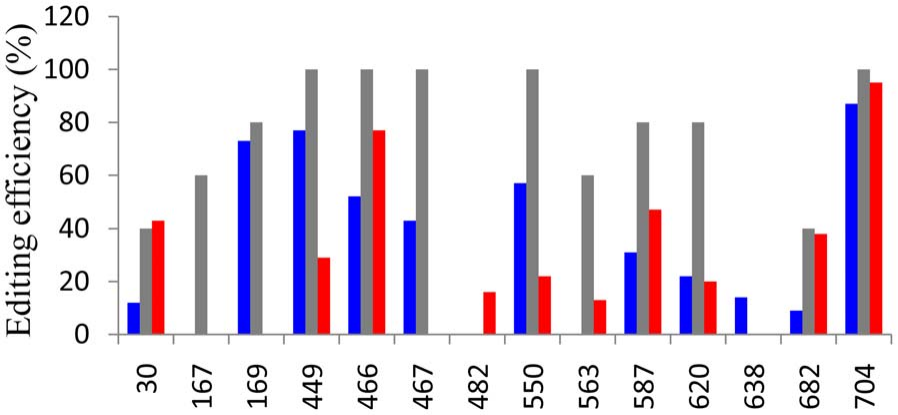
Effect of nearest neighbor mutations on *cox2* mRNA editing. The editing efficiency of different editing sites on *cox2* mRNAs was analyzed for −1 (blue bars) and +1 (red bars) mutants from wheat *cox2* constructs. Gray bars represent the editing status of wild type transcripts. Editing status of C targets was assessed by sequencing of at least 16 independent RT-PCR clones. The results presented are representative from at least two separate experiments. Editing efficiency variations in replicates was lower than 10%.

The editing response was defined as “severely affected” when the editing efficiency was reduced to less than 25% of the wild type, and “slightly affected” when the editing efficiency was comprised between 75% and 25% of the wild type. Individual mutants showed that residues C167, C169, C467, C550, C563 and C620 were severely affected when the neighbor +1 residue was changed, whereas sites C449 and C587 were slightly affected. Residues C30, C466, C682 and C704 were not affected by the +1 mutation (Fig. 1). When the −1 residue was mutated, sites C167, C563 and C682 were severely affected, whereas sites C30, C466, C467, C550, C587 and C620 showed only a moderate reduction of editing. A weak editing efficiency modification for sites C169, C449 and C704 was observed in −1 mutants (Fig. 1).

These results make it possible to define four different recognition patterns based on the role of the +1/−1 nucleotide: (a) +1 dependency for sites C169, C449, C467, C550, and C259, (b) −1 dependency for sites C30, C682 and C77, (c) −1/+1 dependency for sites C167, C563 and C620, (d) no dependency on the nearest neighbor residues for sites C466, C587 and C704.

As previously described, sites C482 and C638 are not considered in this analysis because they were found unedited in electroporation experiments [11]. This observation was confirmed in most of the experiments described here. Unexpectedly, the +1 mutation on site C482 and the −1 mutation on site C638 (Fig. 1) showed a low but significant level of editing for both residues.

### Some editing sites are not autonomous for editing reaction

**Figure 2:**
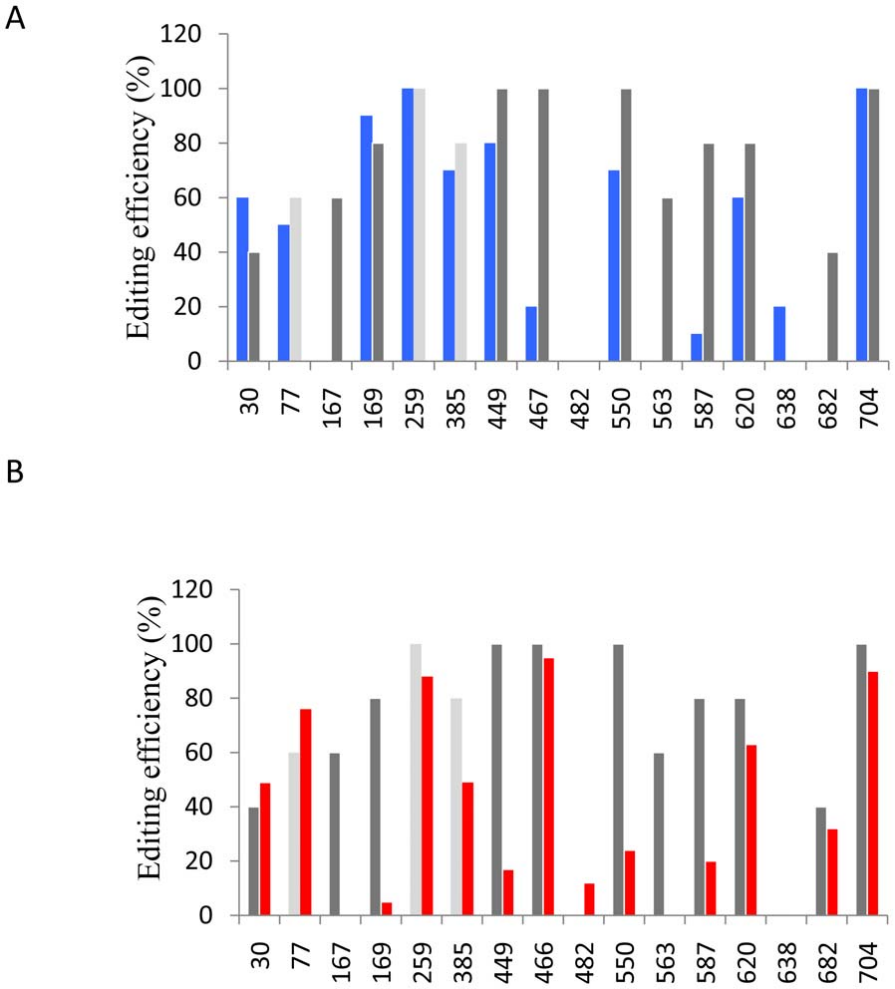
Editing status of *cox2* transcripts carrying combined −1 or +1 mutations. Gray bars represent the editing efficiency on the wild type transcripts. (A) Editing status of mRNA from the combined −1 mutant (blue bars). (B) Editing status of mRNA from the combined +1 mutant (red bars). The sites C77, C259 and C385 not mutated in the −1 or +1 combined constructs are shown by light gray bars. The results presented are representative from at least two separate experiments. Editing efficiency variations in replicates was lower than 10%.

In a second set of experiments, either the −1 or the +1 neighbor residues from all editing sites, with the exception of C77, C259 and C385, were mutated on a single construct. In these mutants, the target residues C466 and C467 are abolished by the −1 and +1 mutation respectively. In both +1 and −1 combined mutant constructs, the overall editing profile and extent of the transcripts were different from the wild type (Fig. 2). Notably, site C385 had a decreased editing efficiency in combined +1 mutant although it was not mutated (Fig. 2B). Comparing the editing efficiency of individual sites in transcripts from the combined mutant with those from single mutants, we observed that sites C167, C169, C449, C482, C550, C638 and C704 presented a similar response. In contrast, sites C30(−1), C620(+1) and C620(−1) showed weaker editing efficiency in transcripts from the single mutant construct than those from combined mutant ones (Table 1). For sites C587(+1) and C587(−1) the situation was the contrary as editing efficiency was higher in combined than individual mutants. Moreover, sites C563(+1) and C682(−1) reached 10% editing efficiency in individual mutants whereas they were not edited in combined constructions. These results indicate that some editing sites are not autonomous and that the editing reaction may be affected by mutations outside of the −16/+6 region. Indeed, the editing profile of *cox2* transcripts from single −1 and +1 mutants (supplemental Fig. S1) indicate that modifications in point mutations may have an impact on the editing status of the mRNA.

**Table 1:** Comparison of RNA editing in single and combined −1 or +1 mutants.

	WT	−1 Mutants		+1 Mutants	
Editing site		single	combined	single	combined
**C30**	40	12	60	43	49
**C167**	60	0	0	0	0
**C169**	80	73	90	0	5
**C449**	100	77	80	29	17
**C466**	100	—	—	77	95
**C467**	100	43	20	—	—
**C482**	0	0	0	16	12
**C550**	100	57	70	22	24
**C563**	60	0	0	13	0
**C587**	80	31	10	47	20
**C620**	80	22	60	20	63
**C638**	0	14	20	0	0
**C682**	40	9	0	38	32
**C704**	100	87	100	95	90

Editing sites are indicated by a C followed by the position of the base on the mature cox2 transcript. Combined mutants present all but C77, C259 and C385 editing sites mutated either at −1 or +1 position in a single construct. Sites C466 and C467 disappeared in the −1 and +1 combined mutants respectively. The numbers indicate the percentage of edited transcripts at the site indicated after sequencing of at least 16 RT-PCR clones.

### Editing is increased in pre-edited molecules

**Figure 3:**
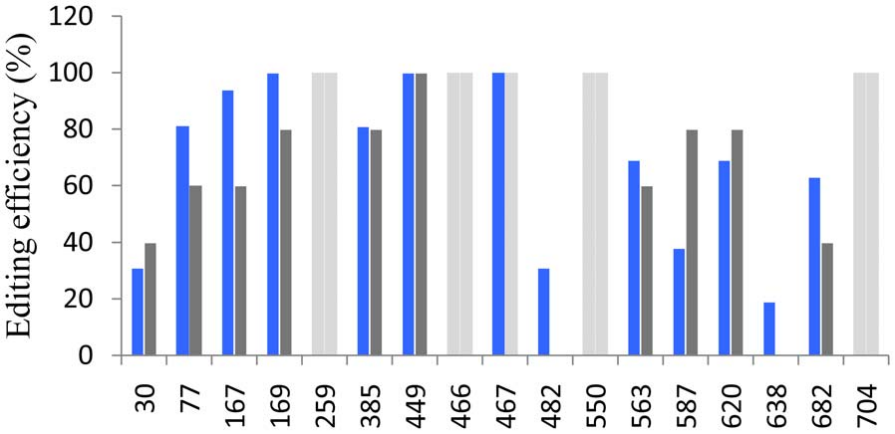
Editing of *cox2* transcripts carrying pre-edited sites. Residues C259, C466, C467, C550 and C704 were changed into T (pre-edited) on the *cox2* transgene. The editing efficiency of the remaining sites was determined by sequencing at least 16 independent RT-PCR clones obtained from spliced products. Gray bars represent the values obtained from wild type transcripts. Blue bars indicate the values from mutated mRNAs. Pre-edited sites are depicted by light gray bars. The results presented are representative from at least two separate experiments. Editing efficiency variations in replicates was lower than 10%.

To verify whether the editing status of one site may have an impact on the editing efficiency of others, we decided to modify the residues that are highly edited in wild type transcripts. For this purpose, sites C259, C466, C467, C550 and C704 were mutated into Ts (pre-edited state). Notably, sites C77, C167, C169, C482, C638 and C682 were more efficiently edited in pre-edited than in wild type mRNAs. The editing efficiency of sites C30, C385, C449, C563 and C620 showed minor differences or none at all. Interestingly, site C587 behaves differently as it was less edited in the pre-edited context than in the wild type one (Fig. 3).

### Editing efficiency is influenced by the status of another site

**Figure 4:**
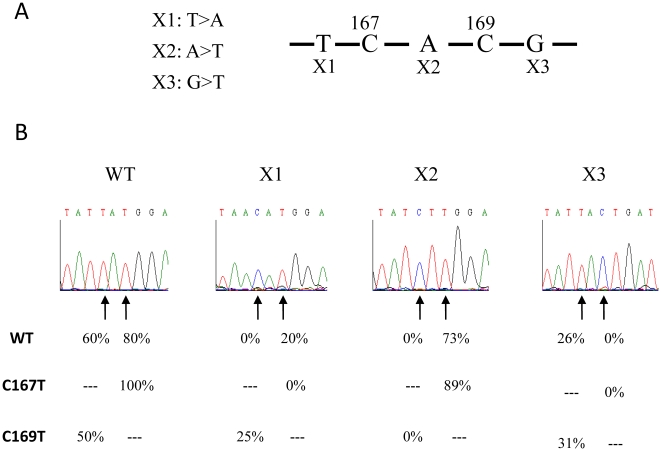
Editing of mutants of overlapping C167 and C169 sites. (A) Sequence encompassing sites C167 and C169. ×1 and ×2 represent the −1 and the +1 mutations of the site C167 respectively. ×2 is also the −1 mutant of site C169. ×3 represents the +1 mutation of the site C169. (B) Chromatopherograms of the sequences surrounding the sites C167 and C169 from different constructs. Arrows indicate the C167 (left) and C169 (right) editing sites. The editing efficiency in wild type and mutant transcripts is indicated below the chromatopherogram. C167T and C169T indicate the presence of a residue T at the place of the editing target C167 and C169 respectively. At least 16 independent RT-PCR clones were sequenced for each construct.

In the case of contiguous editing sites, the C targets are also part of the *cis*-recognition sequence of the neighboring one. The contiguous C167 and C169 editing sites allow the reciprocal influence between editing sites to be studied. For this purpose, different constructs containing either −1 or +1 mutations were expressed in isolated mitochondria. Fig. 4A depicts the different mutations used. As shown above, editing of C167 was strongly affected by −1 or +1 mutation (×1 and ×2). It was reduced about 50% by the mutation at position +3 (×3). In contrast, editing of C169 was not affected by the −1 mutation (×2) but was severely reduced in the mutant + 1 (×3) (Fig. 4B). It should be noted that ×2 represents a +1 modification for C167, but is a −1 mutant with regard to C169. The same mutations were introduced into pre-edited C167T and C169T constructs. Both C167T and C169T presented slight modifications on the editing efficiency of the contiguous residue. The C169T mutant did not affect the editing efficiency of C167 when combined with the ×2 and ×3 mutations compared to the wild type construct. On the other hand, the pre-edited C167T form increased the inhibitory effect of ×1 mutations (Fig. 4B, line C167T). The C169T mutant did not affect the editing efficiency of C167. Similarly, ×1 and ×2 mutations had an effect on C167 editing whatever the C169 status. However, the pre-edited C169T alleviated the editing inhibition when combined with the ×1 mutation (Fig. 4B, line C169T).

## Discussion

RNA editing is a major posttranscriptional process in plant mitochondria where hundreds of particular cytosine residues are changed into uracil in mRNAs to produce functional proteins. Based on *in organello* experiments, it was demonstrated that a region formed by nucleotides −16 to +6 and encompassing the target C constitutes the *cis* element necessary and sufficient to perform the editing reaction [11], [12]. These studies were performed by site-directed mutagenesis on the regions encompassing two sites with different nucleotide sequences, C77 and C259. They also revealed different roles for the individual residues in the −16 to +6 region [11]. Particularly striking is the behavior of mutants affecting the nearest neighbor residue to the C target. Editing of C77(−1) and C259(+1) mutants was completely abolished, indicating that two different responses are possible and that different *trans*-acting factors are involved in their recognition. The length of *cis*-elements and the importance of the nearest residue in RNA editing were confirmed by other approaches [13], [14], [30], [31].

We wondered whether the role of the residue neighboring the C target could be considered as a general recognition mechanism. To obtain more insight into this hypothesis, we systematically changed −1 or +1 residues from the different editing sites on the *cox2* gene and observed the effect on the editing reaction (Fig. 1). We found that the −1 or +1 dependence was not the only way to distinguish the type of recognition for the editing sites. We distinguished four different responses: (a) dependency on the +1 residue for sites, C169, C259, C449, C467 and C550 (this report and [12]); (b) dependency on the −1 residue for sites C30, C77 and C682 (this report and [11]); (c) dependency on both −1 and +1 residues for sites, C167, C563 and C620; (d) no dependency on neighboring residues for sites C466, C587 and C704. Thus, while editing of some sites clearly depends on the identity of the neighboring residue, this situation is not a general rule since some of them are only slightly affected by the mutations or not at all.

The different recognition patterns found for *cox2* mRNA could be an indication of the diversity of *trans* acting factors involved in RNA editing. The discovery of several genes characterized as *trans*-acting factors and involved in plant mitochondrial RNA editing is in agreement with this observation [18], [19], [20], [21], [22], [23], [24], [25], [26], [27], [28]. As some of these proteins recognize several editing sites [19], [20], [23], [25], [26], [27], [28], their different targets might have some features in common. As there is clearly no sequence identity on the *cis*-acting elements [11], the dependency of the editing sites on the +1/−1 nucleotide could constitute a common feature for site recognition by *trans*-acting proteins.

Sites C482 and C638 are efficiently edited in endogenous *cox2* mRNAs but are unedited in electroporation experiments with the wild type construct ([11] and this report). Surprisingly, both sites were found edited in pre-edited constructs and in transcripts from +1 and −1 single mutants, respectively (Supplementary Fig. S1). These observations indicate that the inability to edit C482 and C638 was not due to a deficiency of the experimental model, but rather to the availability of *trans*-acting factors that are probably recruited primarily by other editing sites. One explanation is that mutations increase the binding ability of *trans*-factors for C482 and C638, which conceivably undergo RNA editing later than sites C259 or C704 in wild type transcripts. This may explain some of the results obtained in studies using potato *cox2* genes expressed in heterologous mitochondria [37].

The efficiency of C-to-U conversions for different editing sites in *cox2* mRNAs may be explained by the expression level of specific *trans*-acting factors [28]. Thus, the residues edited most efficiently could be expected to be recognized by abundant *trans*-acting factors. In this regard, several editing sites may compete when recognized by the same limiting *trans-*acting factor. Several reports on chloroplast RNA editing revealed competition between editing sites, suggesting that *trans*-recognition elements may be limiting in the editing reaction [38], [39], [40]. However, our results do not support this idea since the editing efficiency of a particular site can vary in different unrelated mutants. Another possibility is that the affinity of different *trans*-elements, specificity factors and/or deaminases for the respective editing sites are not the same. The dependency on the nearest neighbor residue may be explained by this fact. However, it cannot account for the influence on RNA editing when the mutations are located far from the *cis*-element.

When the −1 (or +1) neighboring residues from a majority of C targets were changed on a single construct, most sites were edited with similar efficiency to that found for single mutants (Table 1). Such a behavior is expected since editing sites are thought to be recognized individually by the editing machinery according to the hit-and-run model [11], [16]. However, this is not the case for sites C30(−1), C620(+1) and C620(−1), which are less efficiently edited in single than in multiple mutants. On the other hand, C587(+1) and C587(−1) showed enhanced editing in single mutants. These editing sites derogate from the postulated autonomy, indicating that the recognition mechanism is more complex than thought and that additional parameters are probably involved. As C30, C587 and C620 are relatively isolated from other editing sites on the transcript, the respective −16/+6 regions were not affected in combined mutants. One can argue that some *cis*-elements may be longer than previously described, or that the editing status of some residues has an impact on the editing ability of others [13], [15], [41]. Moreover, we cannot exclude the hypothesis that the mutations introduced affect RNA editing through changes in the RNA secondary structure.

Structural constraints during RNA processing may indeed explain the variation in editing efficiency observed. A survey of the editing status of the complete set of editing sites for each single mutant either at the −1 or +1 position clearly shows that each construct presents different editing patterns (see Supplementary Fig. S1). This observation is significant since the results from replicate experiments are very reproducible for wild-type or mutant constructs, and are in agreement with the idea that long-range effects on the efficiency of C deamination operate in plant RNA editing.

Transcripts from mutants with sites C259, C466, C467, C550 and C704 changed conjointly into Ts were more efficiently edited than the wild type construct at all sites, except for C587 (Fig. 3). Interestingly, sites C482 and C638 were found edited, as was the case in some −1 and +1 mutants (Figs. 1 and 2). Taken together, these observations support the idea that some C-to-U changes promote the editing reaction of other target Cs, in agreement with the “scanning model” for RNA editing where the editing machinery, once bound to the transcript, searches along the RNA (Fig. 3 and Supplementary Fig. S1) [13], [15], [30]. This is in contrast with the “hit-and-run” model which posits that the choice of editing targets is a stochastic event [11], [41]. However, the majority of *cox2* editing sites analyzed in this report showed an autonomous behavior (Table 1), indicating that no current hypothesis can provide an accurate description of the editing mechanism.

To better understand the effect of pre-edited mutants, we focused on sites C167 and C169 with overlapping *cis-*elements, since they are directly concerned by the C-to-U conversions of the neighboring site (Fig. 4). Interestingly, in the C167(−1) mutant, the conversion of C169 was severely reduced but not in the C167(+1) mutant. The fact that C169 is efficiently edited in mutants where editing of C167 was abolished and in mutants where this residue is pre-edited (C167T, Fig. 4B; see also Supplementary Fig. S1) indicates that the differences observed on C169 were not related to the editing status of the upstream site. In contrast, when site C169 was pre-edited, it counteracted the inhibition of C167 editing observed for the C167(−1) mutant (169TX1, Fig. 4B). One can argue that editing of C167 requires a prior deamination of C169, thus improving the recognition of C167 by the editing machinery. Indeed, an enhanced binding activity of a *trans* acting factor for the edited form of the mRNA has recently been shown [24]. On the other hand, it was reported that the editing of individual sites did not influence the status of the neighboring ones in *atp4* mRNA [41]. In our case, the fact that C167T and C169T pre-edited constructs had little or no effect on the neighboring site in the absence of additional mutation indicates that C-to-U conversion for both sites is not involved in recognition of the nearest neighboring site.

Taken together our results indicate that RNA editing cannot be interpreted solely as the interaction between independent *cis*- and *trans*-elements. It was recently found that altering the mRNA maturation process results in impaired RNA editing [35], [37], [42]. It is thus plausible that the mutations introduced on the transcript may have an impact on different events of RNA maturation, thereby influencing RNA editing. We therefore postulate that transcripts have to be recognized and engaged in the editing process during a coordinated RNA maturation process.

An important conclusion from this study is that some experimental results on RNA editing must be interpreted with caution since this event may be affected by the fate of the mRNA. This may be relevant when studying *trans*-acting factors and may explain why some of them only partially affect the editing reaction [22], [26]. Recently, it was shown that a *trans* acting factor can influence the editing efficiency of two sites different from its primary targets [28]. Similarly, PPR956 is known to reduce the editing efficiency of several sites on a single transcript [18]. As PPR proteins are involved in several organellar gene expression processes [43], the effects induced by mutations have to be carefully investigated in order to discriminate between a direct contribution to RNA editing and a secondary consequence of impaired RNA processing.

## Supporting Information

Figure S1 Editing profile of *cox2* transcripts single mutants. Mutations were performed on the −1 or +1 nearest neighbor residue of the editing target. The number of the editing site corresponds to the position of the C target in the mature transcript, starting from the first nucleotide of the initiation codon. The base changes in −1 and +1 mutant constructs were performed by changing purines by pyrimidines and vice versa. To avoid the introduction of a potential C target, the following changes were performed: A was changed to T, T to A, C to G and G to T depending on the nature of the residue neighbor to the editable C. The results represent the average of at least 16 sequenced RT-PCR clones. The results presented are representative from at least two separate experiments. Editing efficiency variations in replicates was lower than 10%.
(PDF)

Table S1 Oligonucleotide sequences used in the study.
(DOC)
