# Supplementary figures and images for: The RNA Editing Pattern of cox2 mRNA Is Affected by Point Mutations in Plant Mitochondria
Source: PLoS One. 2011 Jun 13;6(6):e20867. doi: 10.1371/journal.pone.0020867 (PMC3113845; doi:10.1371/journal.pone.0020867)

-1 mutant

+1 mutant

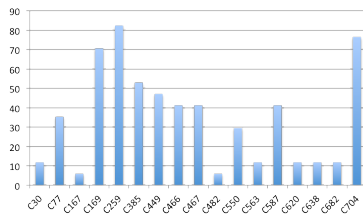

C30

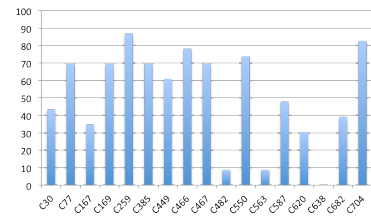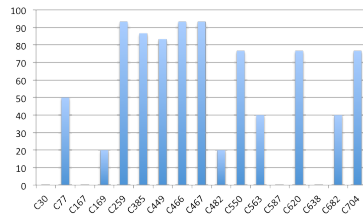

C167

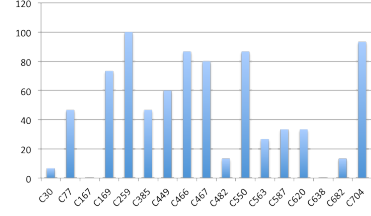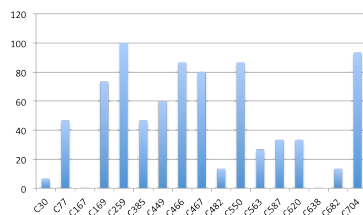

C169

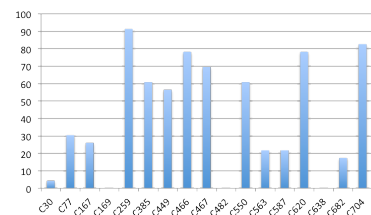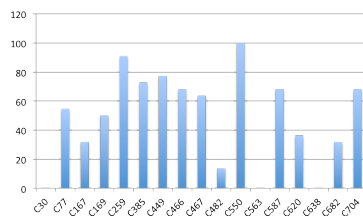

C449

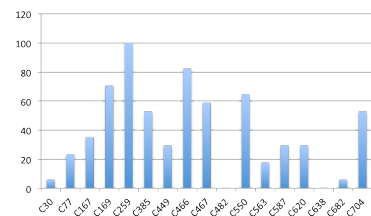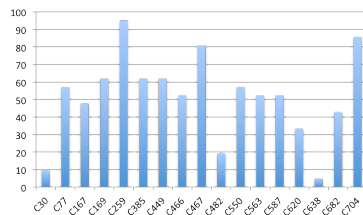

C466

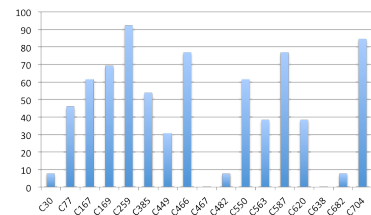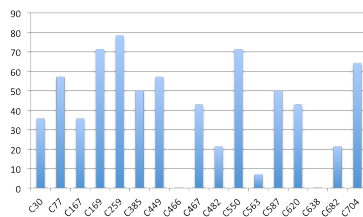

C467

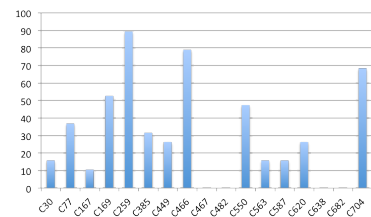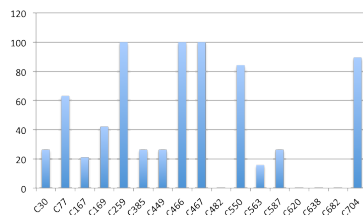

C483

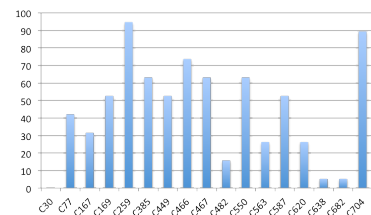

Figure S1

-1 mutant

+1 mutant

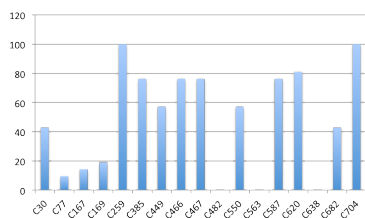

C550

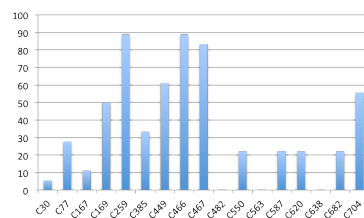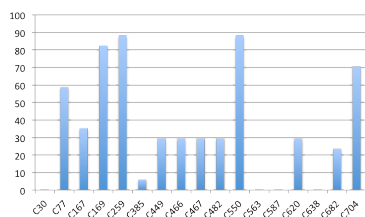

C563

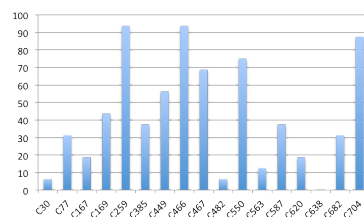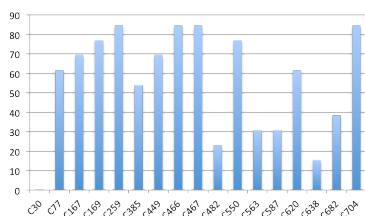

C587

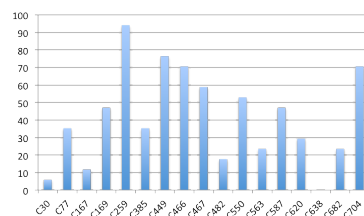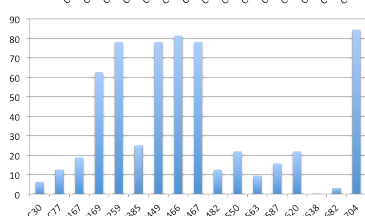

C620

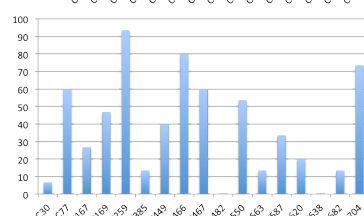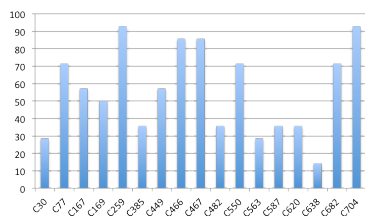

C638

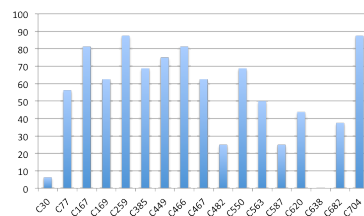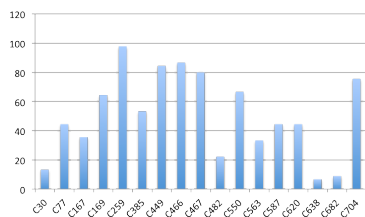

C682

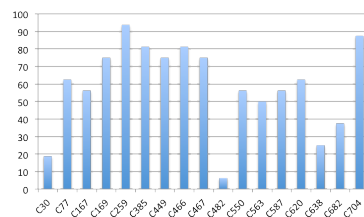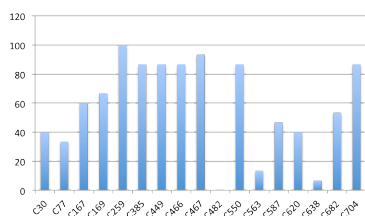

C704

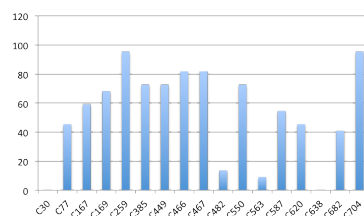

Figure S1

Supplement: Figure S1 — Editing profile of cox2 transcripts single mutants. Mutations were performed on the −1 or +1 nearest neighbor residue of the editing target. The number of the editing site corresponds to the position of the C target in the mature transcript, starting from the first nucleotide of the initiation codon. The base changes in −1 and +1 mutant constructs were performed by changing purines by pyrimidines and vice versa. To avoid the introduction of a potential C target, the following changes were performed: A was changed to T, T to A, C to G and G to T depending on the nature of the residue neighbor to the editable C. The results represent the average of at least 16 sequenced RT-PCR clones. The results presented are representative from at least two separate experiments. Editing efficiency variations in replicates was lower than 10%. (PDF) [file pone.0020867.s001.pdf]
