# Supplementary material for: The RNA Editing Pattern of cox2 mRNA Is Affected by Point Mutations in Plant Mitochondria
Source: PLoS One. 2011 Jun 13;6(6):e20867. doi: 10.1371/journal.pone.0020867 (PMC3113845; doi:10.1371/journal.pone.0020867)
Supplement: Table S1 — Oligonucleotide sequences used in the study. (DOC) [file pone.0020867.s002.doc]

**TABLE S1.** Oligonucleotide sequences used in the study.

| **Mutated site** | **Oligonucleotides (only the sense sequence is shown)** |
| --- | --- |
| C30(+1) | tcatgtcgattc**g**tcacaatcgc |
| C167(+1) | gttttcgtatc**t**cggatgttggtt |
| C169(+1) | ttttcgtatcac**t**gatgttggttc |
| C449(+1) | tgacagttatac**t**attccagaaga |
| C466(+1) | cagaagatgatc**g**agaattgggt |
| C467(+1) | agaagatgatcc**t**gaattgggtc |
| C482(+1) | attgggtcaatc**t**cgtttattagaa |
| C550(+1) | tgattgtaacac**g**cgctgatgta |
| C563(+1) | cgctgatgtacc**a**catagttggg |
| C587(+1) | tgtaccttcctc**t**ggtgtcaaat |
| C620(+1) | acctggtcgttc**t**aatcttacct |
| C638(+1) | tacctccatctc**t**gtacaacgag |
| C682(+1) | gcagtgagattc**t**tggaactaat |
| C704(+1) | tcatgcctttac**t**cctatcgtcg |
| C30(-1) | tcatgtcgat**a**cctcacaatcgc |
| C167(-1) | ggttttcgta**a**cacggatgtt |
| C169(-1) | gttttcgtatc**t**cggatgttggtt |
| C449(-1) | tgacagttat**t**cgattccaga |
| C466(-1) | cagaagatga**a**ccagaattgggt |
| C467(-1) | agaagatgat**g**cagaattgggtc |
| C482(-1) | aattgggtcaa**a**cacgtttatta |
| C550(-1) | tgattgtaac**t**cccgctgatgta |
| C563(-1) | cgctgatgta**g**ctcatagttggg |
| C587(-1) | tgtaccttcc**a**caggtgtcaaat |
| C620(-1) | acctggtcgt**a**caaatcttacct |
| C638(-1) | ttacctccatc**a**cggtacaaccg |
| C682(-1) | gcagtgagat**a**cgtggaactaat |
| C704(-1) | tcatgccttt**t**cgcctatcgtcg |
| 259T | tcgaaattatt**t**ggaccatattt |
| 466-467T | cagaagatgat**tt**agaattgggtc |
| 550T | tgattgtaaca**t**ccgctgatgta |
| 704T | tcatgccttta**t**gcctatcgttcg |
| 167T | ggttttcgtat**t**acggatgttgg |
| 167TX1 | ggttttcgta**at**acggatgttgg |
| 167TX2 | ggttttcgtat**tt**cggatgttgg |
| 167TX3 | ggttttcgtat**t**ac**t**gatgttgg |
| 169T | ttttcgtatca**t**ggatgttggtt |
| 169TX1 | ttttcgta**a**ca**t**ggatgttggtt |
| 169TX2 | ttttcgtatc**tt**ggatgttggtt |
| 169TX3 | ttttcgtatca**tt**gatgttggtt |
| X1+X2 | C-167 + ggttttcgtaac**t**cggatgttgg |
| X1+X2+X3 | C-167 + ggttttcgtaac**t**cggatgttgg + ggttttcgtaactc**t**gatgttgg |
